# Prevalence of Latent Tuberculosis Infection Among Rheumatology Patients and Management Practices in the United Arab Emirates: A Single-Center Retrospective Cohort Study

**DOI:** 10.7759/cureus.50581

**Published:** 2023-12-15

**Authors:** Shamma Al Nokhatha, Fatima AlKindi, Maryam Alfalasi, Merna Abdelsalhen, Fatima AlKhyeli, Ahmad R Alsaber

**Affiliations:** 1 Rheumatology, Tawam Hospital, Al Ain, ARE; 2 Internal Medicine, College of Medicine and Health Sciences, United Arab Emirates University, Al Ain, ARE; 3 Internal Medicine, Tawam Hospital, Al Ain, ARE; 4 College of Business and Economics, American University of Kuwait, Kuwait, KWT

**Keywords:** united arab emirates (uae), indeterminate, immunosuppression, latent tuberculosis infection, interferon-gamma release assay (igra)

## Abstract

Introduction

Prior to immunosuppression, rheumatology patients are routinely screened for latent tuberculosis (TB) infection using interferon-gamma release assays (IGRAs). Variability in the management of latent and indeterminate IGRA results across institutions limited long-term outcome data. A retrospective study was conducted at Tawam Hospital, United Arab Emirates, to investigate the incidence and management protocols associated with positive and indeterminate IGRA results, as well as TB infection, among patients with rheumatic conditions.

Methods

A single-center retrospective observational study was performed at Tawam Hospital, Abu Dhabi, UAE. Ethical approval for this study was obtained from the Tawam Human Research Ethics Committee. Laboratory records and the hospital's electronic medical system were used to obtain information about IGRA results over a 12-year period (April 2010-April 2022). The hospital's electronic medical system was used to obtain patient information and subsequent management approaches of positive and indeterminate IGRAs. Moreover, long-term follow-up data were collected to determine the risk of TB reactivation in the cohort.

Results

We found a total of 1,012 positive and 223 indeterminate IGRA test results within the 12-year period. Within the rheumatology department, 123 positive and 39 indeterminate IGRA results were identified. In the indeterminate IGRA group, the majority were women (n = 24, 61.5%) and UAE nationals (n = 22, 56.4%), and their mean age was 38.6 years. Systemic lupus erythematosus was the most prevalent rheumatologic condition (n = 21, 53.8%). Thirteen (33.3%) were on disease-modifying anti-rheumatic drugs (DMARDs) and 26 (66.7%) were on corticosteroids during IGRA testing. A total of eight patients (20.5%) received anti-TB medications. In the positive IGRA group, the mean age was 55.7 years and the female-to-male ratio was 3:1. The most common rheumatologic condition was rheumatoid arthritis (n = 69, 56%). Sixty-five (52.8%) patients were on conventional DMARDs, 43 (34.9%) were on corticosteroids during IGRA testing, and 74 (60%) received anti-TB medications. Two cases (1.6%) of active TB infections were detected among patients with positive IGRA tests, both of whom were receiving anti-tumor necrosis factor alpha inhibitor treatment in combination with methotrexate. No cases of active TB infection were observed in the indeterminate IGRA group.

Conclusion

Long-term data on the risk of TB activation in positive and indeterminate IGRA results for rheumatological conditions are low. It is recommended to reassess the choice of using anti-TNF-α, with a positive IGRA result if no other feasible alternatives can be offered. Our findings stress the importance of age, underlying diseases, and immunosuppressive treatments in interpreting IGRA results and guiding patient management. A large multicenter study is needed to understand the differences and outcomes of such patients in TB endemic and nonendemic geographical areas.

## Introduction

Tuberculosis (TB) is a major health problem. The World Health Organization has defined latent tuberculosis infection (LTBI) as a state of persistent immune response to stimulation by Mycobacterium tuberculosis antigens without evidence of clinically manifested active TB [1]. TB bacteria can remain dormant for years, and in 10% of individuals with LTBI, the infection may progress to active TB. In half of these individuals, the progression occurs within the first two years of acquiring the infection, and in the other half, progression occurs after two years. The overall prevalence rates of LTBI in the Middle East and North African regions are 41.78% and 43.81% of the adult population, respectively [2].

Many immunosuppressive drugs are potent T-cell inhibitors that can impair the interferon response. Studies have shown that patients undergoing immunosuppression treatment, particularly with regard to tumor necrosis factor alpha (TNF-α), have an increased risk of TB; for example, the relative risk is 29.3 for patients taking adalimumab and 18.6 for those taking infliximab [3,4]. Furthermore, a study by the British Society for Rheumatology Biologics Register reported a 3- to 4-fold higher rate of TB with infliximab (144 events/100,000 person-years) and adalimumab (136/100,000 person-years) in comparison with etanercept (39/100,000 person-years) group [5].

Screening for LTBI and treating individuals who test positive are the cornerstones of TB prevention and are particularly important in high-risk patients, especially those with autoimmune disorders [3].

This study aimed to evaluate the frequency of positive and indeterminate interferon-gamma release assay (IGRA) tests, the management approach, and the risk of TB reactivation in rheumatologic patients at a tertiary hospital in the United Arab Emirates (UAE).

## Materials and methods

A single-center retrospective observational study was performed at Tawam Hospital, Abu Dhabi, UAE. Ethical approval for this study was obtained from the Tawam Human Research Ethics Committee. The department record system was searched to identify all patients on immunosuppression therapy for recruitment in the study. All adult patients (aged ≥16 years) attending the rheumatology clinic during a 12-year period (October 2010-April 2022) were enrolled. Those with positive and indeterminate IGRA testing were included in the analysis. Patients with negative IGRA results, those lost to follow-up, and those with active TB at the time of diagnosis of an autoimmune disease were excluded. A chart review was performed to gather demographic, radiological, and clinical data and management outcomes of patients with positive and indeterminate IGRA tests. The need for infectious disease (ID) referral and the use of anti-TB medications were evaluated. Moreover, long-term follow-up data were collected to determine the risk of TB reactivation in the cohort.

Statistical analysis

Descriptive data are expressed as mean ± standard deviation (SD), median (range), or number and frequency, as applicable. Quantitative variables are expressed as mean and SD or median and quartile. For the comparison of the groups, the Wilcoxon or Mann-Whitney test for the means was used depending on the normality test. Univariate and multivariate logistic regression analyses were performed to identify the factors correlated with positive and indeterminate tests. The significance level was set at P < 0.05. Statistical analysis was performed using the Jamovi 2.3.21.0 program (Jamovi Project, https://www.jamovi.org). RStudio (Version 2023.03.0+386) and R (Version 4.2.3) were employed for data cleaning and logistic regression modeling.

## Results

A total of 1,012 positive and 223 indeterminate LTBI tests were identified in the 12-year period, of which 39 indeterminate and 123 positive results met the inclusion criteria.

Indeterminate IGRA results

Thirty-nine rheumatologic patients had indeterminate IGRA results. Twenty-four (61.5%) were women and 22 (56.4%) were UAE nationals, and their mean age was 38.6 years (SD=17.1). The predominant rheumatologic conditions in the cohort were systemic lupus erythematosus (SLE) (21, 53.8%), rheumatoid arthritis (RA) (four, 10.3%), psoriatic arthritis (PSA) (four, 10.3%), and small vessel vasculitis (five, 12.8%). The median duration of rheumatologic disease since diagnosis was 7.75 years (6 months-15 years).

Conventional synthetic disease-modifying antirheumatic drugs (csDMARDs) were used in 13 (33%) of the patients. Corticosteroids were used in 26 (66.7%), and the mean prednisolone dose at the time of the IGRA test was 91 ± 241 ­­mg. Moreover, four (10.3%) of the patients were covered with biologics at the time of the IGRA test for various reasons, including switching immunosuppression, in-patient work-up owing to symptoms, contact with patients having active TB, and periodic medical check-ups, or for no reason at all. Table 1 provides a comparison between the characteristics of patients with positive and indeterminate IGRA results.

**Table 1:** Characteristics of patients with positive and indeterminate interferon-gamma release assay (IGRA) testing csDMARDs, conventional disease-modifying antirheumatic drugs; EGPA, eosinophilic granulomatosis with polyangiitis; FMF, familial Mediterranean fever; GPA, granulomatosis with polyangiitis; MCTD, mixed connective tissue disease; PSA, psoriatic arthritis; RA, rheumatoid arthritis; SLE, systemic lupus erythematosus; SPA, spondylarthritis; TB, tuberculosis.

Characteristics	Positive IGRA	Indeterminate IGRA
Patients, n	123	39
Female, n (%)	90 (73.1)	24 (61.5)
Male, n (%)	33 (26.8)	15 (38.5)
Mean age, years	55.7	38.6
National, n (%)	78 (63.4)	22 (56.4)
Types of rheumatology diseases, n (%)		
SLE	17 (13.8)	21 (53.8)
RA	69 (56)	4 (10.3)
PSA	10 (8.1)	4 (10.3)
Bechet	6 (4.8)	0
Vasculitis	11 (8.9)	5 (12.8)
SPA	4 (3.3)	0
Others	6 (4.9)	5 (12.8)
FMF	1	1
Dermatomyositis or polymyositis	2	0
Scleroderma	1	0
Sjögren syndrome	2	1
Sarcoidosis	0	1
Still disease	0	1
MCTD	0	1
Previous TB infection and treatment, n	3	0
Immunosuppression medications and biologics, n (%)		
csDMARDs with and without hydroxychloroquine	65 (52.8)	13 (33.3)
Methotrexate	41 (33.3)	4 (10.3)
Sulfasalazine	8 (6.5)	1(2.6)
Leflunomide	1 (0.8)	0
Azathioprine	10 (8.1)	2 (5.1)
Mycophenolate mofetil	5 (4.1)	6 (15.4)
Anti-TNF therapy		
Adalimumab	5 (4.1)	0
Etanercept	2 (1.6)	2 (5.1)
Infliximab	1 (0.8)	2 (5.1)
Interleukin 17 inhibitors		
Ixekizumab	1 (0.8)	0
JAK-inhibitors		
Tofacitinib	1 (0.8)	0
Others		
Abatacepts	1 (0.8)	0
Rituximab	2 (1.6)	0
Corticosteroid ≥ 15 mg	23 (18.7)	15 (38.5)
Concomitant therapy	40 (32.5)	14 (35.9)
Median year of rheumatologic condition diagnosis, years (range)	18.75 (0.5-37)	7.75 (6 months to 15 years)
No smoking, n (%)	84 (68.2)	27 (69.2)
IGRA test median, years (range)	6.75 (0.5-13)	6.25 (6 months to 12 years)
Repeat IRGA test results, n (%)		
Repeated	28 (22.8)	14 (35.9)
Positive	21 (17.1)	3 (7.7)
Indeterminate	1 (0.8)	7 (17.9)
Negative	6 (4.9)	4 (10.3)
Not repeated	95 (77.2)	25 (64.1)
TB infection on follow-up	2 (1.6)	0
TB lymphadenitis	1	0
TB pulmonary/pleural	1	0
Anti-TB medications, n (%)	74 (60.2)	8 (20.5)
Active TB infection	5 (3 with a history of active TB prior to their autoimmune diagnosis and 2 after autoimmune diagnosis)	0
Latent TB	69	8

In almost one-third of the patients (n = 14, 35.9%), the IGRA tests were repeated, which revealed indeterminate results in seven patients, negative results in four patients, and positive results in three patients. Chest radiographs were acquired for two-third of patients (n = 26, 66.7%), and only one-third of patients (n = 13, 33%) required chest computed tomography (CT). Additionally, ID consultations were sought for 43.6% (n = 17) of the cases. A total of eight (20.5%) patients received anti-TB medications owing to a diagnosis of LTBI (isoniazid monotherapy (INH) and vitamin B6 for nine months). The majority of patients were on maintenance immunosuppression medications and biologics during the follow-up period without evidence of reactivation of TB infection.

Positive IGRA results

Positive IGRA results were obtained in 123 rheumatologic patients. Their mean age was 55.7 years (SD=16.5), 78 patients (63.4%) were UAE nationals, and the female-to-male ratio was 3:1. The most common rheumatologic conditions were RA (n = 69, 56%), SLE (n = 17, 13.8%), PSA (n = 10, 8.1%), and Bechet disease (n = 6, 4.8%). csDMARDs were used in 65 (52.8%) of the patients. Corticosteroids were used in 43 (34.9%), and the mean dose at the time of the IGRA test was 40 ± 166 mg.

**Figure 1:**
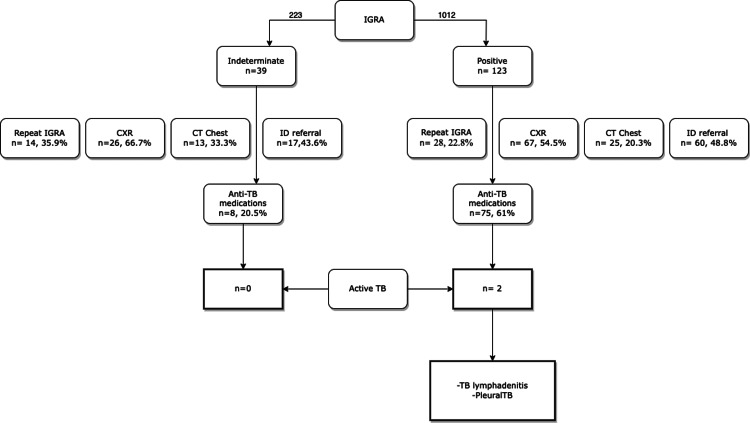
Post-IGRA screening of rheumatology patients in Tawam Hospital, 2010-2022 CT, computed tomography; CXR, chest x-ray; ID, infectious disease; IGRA, interferon-gamma release assay; TB, tuberculosis

The IGRA test was repeated in 28 (22.8%) patients, chest radiographs were acquired for half of the patients (n = 67, 54.5%), and chest CT was performed in one-fifth of the cases (n = 25, 20.3%). ID consultation was required for sixty patients (48.8%), and five patients had active TB infection. Seventy-four (60%) of the patients were treated with anti-TB medications, including those with active TB infection (n = 5) and those with LTBI (n = 69). Of note, three patients had a previous history of treatment for TB infection. These patients did not receive repeat courses despite positive IGRA testing, and there was no evidence of active infection during follow-up. The other two patients with active TB were on TNFα inhibitor (Figure 1).

In univariate analysis for RA, current prednisolone use and prednisolone dose ≥ 15 mg were independently associated with positive IGRA results (P < 0.05). In multivariate analysis, male sex (odds ratio [OR] = 7.27; 95% confidence interval [CI]: 2.24-27.83, P = 0.002) and current prednisolone use (OR = 4.31; 95% CI: 1.2-16.14, P = 0.026) were associated with positive IGRA results (Table 2).

**Table 2:** The comparison between positive and indeterminate conditions. The independent variables or predictors include age, sex, whether the individual has rheumatoid arthritis, systemic lupus erythematosus, psoriatic arthritis, vasculitis, current use of prednisolone, use of corticosteroid (≥15 mg), and use of biologics

Dependent: type	Positive	Indeterminate	OR (univariable)	OR (multivariable)
Age, mean (SD)	55.7 (16.5)	38.6 (17.1)	0.94 (0.91-0.96, P < 0.001)	0.96 (0.92-0.99, P = 0.007)
Sex				
Female	90 (78.9)	24 (61.5)	-	-
Male	33 (68.8)	15 (38.5)	1.70 (0.79-3.62, P = 0.168)	7.27 (2.24-27.83, P = 0.002)
Rheumatoid arthritis	69 (56)	4 (10.3)	0.09 (0.03-0.24, P < 0.001)	0.44 (0.09-2.17, P = 0.307)
Systemic lupus erythematosus	17 (13.8)	21 (53.8)	7.27 (3.27-16.70, P < 0.001)	6.51 (1.53-32.67, P = 0.015)
Psoriatic arthritis	10 (8.1)	4 (10.3)	1.29 (0.34-4.13, P = 0.681)	2.34 (0.37-14.75, P = 0.360)
Vasculitis	11 (8.9)	5 (12.8)	1.50 (0.45-4.43, P = 0.482)	0.74 (0.13-4.08, P = 0.729)
Current prednisolone	43 (34.9)	26 (66.7)	3.72 (1.77-8.18, P = 0.001)	4.31 (1.20-16.14, P = 0.026)
Corticosteroid, ≥ 15 mg	23 (18.7)	15 (38.5)	2.72 (1.23-5.98, P = 0.013)	0.88 (0.24-3.23, P = 0.840)
Biologic /small molecules	13 (10.6)	4 (10.3)	0.76 (0.21-2.25, P = 0.650)	1.59 (0.31-6.99, P = 0.552)

## Discussion

In this study, 123 positive and 39 indeterminate IGRA testing results were obtained in rheumatologic patients over a period of 12 years, with prevalence rates of 12.2% and 17.5%, respectively, across all subspecialties. These rates are much lower than the rate for the general population in the Middle East (41.78%) [2]. The prevalence rate of LTBI in patients with rheumatic diseases differs from country to country, with 20.4% in India [6], 21.6% in Morocco [7], and 29.5% in Brazil [6]. These discrepancies in the outcomes could be attributed to variations in the study design and the impact of immunosuppression and corticosteroid usage on interpretation [6,7].

This study found an inverse association between old age and positive IGRA results. With every one-year increase in age, the odds of being in the positive IGRA group decreased by approximately 6% in the univariable analysis (OR = 0.94, P < 0.001) and by approximately 4% in the multivariable analysis (OR = 0.96, P = 0.007). This finding is in line with some studies suggesting a strong association between old age (≥65 years of age) and indeterminate results [8,9].

In our cohort, patients with RA were observed to exhibit higher positive IGRA results, which agrees with previous research studies. RA itself increased the risk of TB infection among patients compared with the general population, and this association was independent of the use of biologics [10,11]. In multivariate analysis, no significant association was found between RA and positive and indeterminate IGRA results (OR = 0.44 (0.09-2.17, P = 0.307). In contrast, SLE was more common in the indeterminate group, which is similar to reports in the literature. Maharani et al. [12] demonstrated that active disease status resulted in indeterminate IGRA results in 12.66% of the patients in the SLE group, which was validated by another study [13]. In the present study, the disease activity status was not included as it was out of the study scope.

Regarding the subsequent management approach, only half of the positive IGRA group had a chest x-ray (CXR), and approximately one-third proceeded to chest CT. Although there is limited evidence for the usefulness of CXR in screening, it is still recommended to improve the specificity. However, chest CT is considered more effective in identifying active TB in patients with a positive IGRA test, particularly if deemed necessary [14,15].

Owing to uncertainties in the subsequent steps following an indeterminate IGRA result, repeating the test is recommended. Nearly one-third of the patients in our cohort (n = 14, 35.9%) had a repeat test. Throughout the study duration, two-thirds of the patients had a CXR and one-third required chest CT. It is important to note that clear guidelines regarding the use of either approach are lacking unless the repeat test is positive and when necessary [16].

Immunosuppressants and corticosteroids can worsen an abnormal immune response. In this study, two factors contributed to the likelihood of obtaining an indeterminate result. Alongside a diagnosis of SLE, the utilization of corticosteroids negatively influenced the outcome of the IGRA test. Two third (n=14) of the SLE patients in the indeterminate group were treated with corticosteroids (doses of 15 mg, or >15 mg), which could explain the indeterminate result. Previous studies have documented that corticosteroid use increases the likelihood of having an indeterminate result, but some studies have reported conflicting outcomes [17-19]. Nevertheless, in this study, no differences were seen in the use of corticosteroid doses ≥15 mg between the two studied groups, which could be a consequence of the small sample size.

With long-term follow-up, two cases (1.6%) of active TB infections were identified in patients with positive IGRA tests. All patients were treated with TNFα inhibitor (Adalimumab, 40 mg every two weeks) in conjunction with methotrexate (10-20 mg, weekly) for a duration of 208 and 312 weeks. In contrast, there were no cases of active TB in the indeterminate group.

In any case, TNF plays an important role in the host’s response to infection, by maintaining the integrity of the granuloma that forms as a result of the infection. Thus, the use of TNF antagonists causes disruption of the granuloma integrity resulting in mycobacterial growth and activation. This supports our findings, while data from clinical trials reported negligible TB reactivation in non-anti-TNFa biologic [6,20].

Infectious disease standards of care

Screening for LTBI using the IGRA or tuberculin skin test (TST) is recommended in high-risk patients, including HIV-positive, solid organ transplant, stem cell transplantation, and immunocompromised patients receiving biological therapy, especially TNFa antagonists [21]. The IGRA test is better than the TST in previously BCG-vaccinated immunosuppressed patients, with an estimated sensitivity of 67%-75% and specificity of 93%-99% [22,23]. A detailed medical history of signs and symptoms suggestive of active TB infection, history of exposure to patients with active TB, travel or migration from endemic areas, type of immunosuppression medications, and medical comorbid conditions should be obtained. LTBI is diagnosed based on positive IGRA/TST testing, negative CXR or chest CT, and no evidence of active TB infection [21,24]. Consultation with the ID team is required for LTBI treatment in immunosuppressed patients.

For several years, isoniazid (5 mg/kg, 300 mg/d) (INH) supplemented with pyridoxine (vitamin B6) for nine months was the standard treatment for latent TB. However, this treatment is associated with a risk of hepatic injury and noncompliance with the therapy duration. Regular monitoring for hepatotoxicity is critical in patients receiving INH therapy, and the medication should be withheld whenever indicated. INH-related hepatotoxicity is defined as an increase in liver aminotransferases >5 times the upper normal limit or >3 times the normal limit with symptoms. Alternatively, a 4-month course of rifampin carries a lower risk for liver injury than INH [25]. Nevertheless, rifampin is a potent cytochrome P450 inducer and can accelerate the metabolism of some immunosuppressive agents (calcineurin inhibitors, including cyclosporine and tacrolimus). Hence, drug-drug interactions should be monitored. Three months of daily INH plus rifampin has also been approved for LTBI therapy [26].

Sterling et al. reported comparable effectiveness for directly observed, once-weekly therapy with rifapentine plus INH for three months and a self-administered nine-month course of daily INH [27]. The three-month therapy was well tolerated, with lower rates of adverse events and higher compliance. The National Tuberculosis Controllers Association and Centers for Disease Control and Prevention 2020 LTBI treatment guidelines recommend the use of rifamycin-based regimens, including three months of once-weekly INH plus rifapentine, four months of daily rifampin, and three months of daily INH plus rifampin. These are the preferred recommended regimens because of their effectiveness, safety, and high treatment completion rates. Alternative LTBI therapeutic regimens are 6 or 9 months of daily INH [24].

Consensus is lacking on the safe period for starting biological or immunosuppressive therapy in patients with LTBI. Some advocate ruling out active TB infection and commencing LTBI treatment 3 weeks to 2 months prior to the initiation of immunosuppressive medications [21]. Potential drug-drug interactions should be monitored. How frequently patients undergoing long-term biological or immunosuppressive therapy should be screened for LTBI is not well established. Immunosuppressed patients who were treated for LTBI previously will require further evaluation and ID referral for optimal risk assessment and for determining whether repeat LTBI therapy is needed [21].

Rheumatology standards of care

According to the 2018 guidelines of the British Society of Rheumatology [28], all patients must be screened for TB before commencing treatment with biologics. This screening involves a clinical examination, a CXR, and either a TST or an IGRA test. If a positive result is obtained for LTBI, the patient must begin treatment at least 1 month before starting biologic therapy, with monitoring at 3-month intervals. Etanercept should be the first line of treatment for patients who require anti-TNF therapy and are at a high risk of TB reactivation because anti-TNF monoclonal antibody medications (particularly adalimumab and infliximab) have a higher risk of TB reactivation than etanercept [28].

The European Alliance of Associations for Rheumatology [14] recommends that all patients be screened for LTBI before starting treatment with biologic DMARDs or targeted synthetic DAMRDs. Additionally, if a patient is deemed to be at a high risk owing to factors such as alcohol abuse, smoking, living with people who have TB, or living in endemic countries, screening should also be performed if the patient is considering csDMARDs and/or glucocorticoids. No consensus exists on the recommended dose or duration for glucocorticoid usage, but based on previous studies, screening should preferably be done if the glucocorticoid dose is ≥15 mg/d and if the treatment period exceeds four weeks. This screening can be accomplished via CXR and IGRA. However, guidance on how frequently the test should be performed or when it should be repeated has not been updated. The American College of Rheumatology recommends annual testing for high-risk patients who live or travel to endemic countries [14,29]. However, such recommendations must be regularly updated, especially as new medications are developed. Management is based on international guidelines and the most often utilized regimens mentioned previously.

This is the first study in the UAE to address the IGRA test results in rheumatologic conditions over the course of more than one decade. Some of the limitations were a small sample size of patients, the single-center experience, and a lack of a comparative group.

## Conclusions

Long-term data on the risk of TB activation in positive and indeterminate IGRA results for rheumatological conditions are low. It is recommended to reassess the choice of using anti-TNF-α, with a positive IGRA result if no other feasible alternatives can be offered. Our findings stress the importance of age, underlying diseases, and immunosuppressive treatments in interpreting IGRA results and guiding patient management. A large multicenter study is needed to understand the differences and outcomes of such patients in TB endemic and nonendemic geographical areas.
